# World Cafés as a method of identifying cross-sectoral policy solutions to social determinants of health equity in Australia

**DOI:** 10.1093/heapro/daag135

**Published:** 2026-09-04

**Authors:** Toby Freeman, Connie Musolino, Fran Baum

**Affiliations:** Stretton Health Equity, Adelaide University, Kaurna, Adelaide SA 5005, Australia; Stretton Health Equity, Adelaide University, Kaurna, Adelaide SA 5005, Australia; Stretton Health Equity, Adelaide University, Kaurna, Adelaide SA 5005, Australia

**Keywords:** qualitative research methods, social determinants of health, health promotion, health equity, multi-disciplinary

## Abstract

World Cafés are a method to promote inclusive, constructive dialogue to identify solutions to large scale problems. These attributes make World Cafés a potentially valuable tool for multi-sectoral dialogue to identify solutions to health promotion issues such as addressing social determinants of health inequities. However, there are few accounts of World Cafés being used to bring disparate sector knowledges together to address common problems. We sought to test how the World Café method could aid bringing together multi-disciplinary and multi-sector expertise to identify solutions to reduce increasing health inequities in Australia. We conducted World Cafés in each Australian state and territory (*N* = 8) with a total of 162 participants from academia, government, and civil society. Participants were drawn from sectors including health, education, housing, urban planning, economics, and employment, and from organizations representing Aboriginal and Torres Strait Islander peoples, people with a disability, multicultural communities, youth, and older people. We found different sector participants readily found common ground and collaborated to identify potential public policy solutions to health inequities, generating deep discussion on structural drivers that would not be possible without bringing together these sector knowledges. This upstream focus is likely to identify opportunities to make meaningful changes to create healthier, more equitable societies. Participants reported valuing participation in the World Cafés, making the research more of an exchange compared to other data collection methods. We would suggest World Cafés are a valuable addition to a research toolkit for seeking structural solutions to health promotion issues.

Contribution to Health Promotion

- This article provides an account of the implementation of the World Café method on a health promotion topic.
- World Cafés are a valuable research method for bringing together expertise from a wide range of sectors to identify solutions to addressing the social determinants of health equity.
- The method allowed deep discussions of upstream health promotion and was valued by participants for the chance to discuss shared overarching drivers of problems across sectors, and to build professional networks.

## Introduction

Population health and health equity are driven by the social determinants of health, such as the distribution of income and wealth, housing, neighbourhoods, and provision of services (Commission on the Social Determinants of Health 2008, Marmot and Allen 2014). Government policy is a powerful instrument to shape the availability, nature, and equitable spread of these social determinants (Baum and Friel 2017, Baum *et al*. 2018, Freeman *et al*. 2020). Thus, creating supportive environments for health and equity requires policy solutions across many sectors (World Health Organization 2023). This requires bringing together knowledge and expertise from many disciplines and sectors to identify policy solutions to effectively and feasibly improve social determinants of population health and equity. Crossing discipline and sector divides can be difficult for policy makers, academics, and civil society organizations; however, all of whom risk being constrained to siloed working by numerous institutional drivers (Flavel *et al*. 2022, Carrad *et al*. 2023, Newman 2024). In Australia, persistent and rising health inequities (Flavel *et al*. 2022a, Timonin *et al*. 2025) highlight the urgency of finding ways to overcome these institutional barriers. We sought to investigate the potential for a research method, World Cafés, to bridge these sectoral divides and allow different experts to come together to develop solutions to a complex health promotion issue.

### What is a World Café?

World Cafés are a method developed by Juanita Brown and David Isaacs in 1995 to facilitate conversations with large groups on complex topics (Brown 2005). Brown and Isaacs came from business and organizational learning backgrounds, and drew from the organizational learning tradition of Appreciative Inquiry, which focuses on the transformational and relational process of studying human organization with a strengths-based approach (Whitney and Trosten-Bloom 2010). The aim of the World Café method was to promote inclusive, constructive dialogue to better identify solutions to large scale problems, with particular uptake by business organizations (Brown 2005). It has since been adapted as a qualitative research method for the social sciences (Löhr *et al*. 2020). While there are similarities between World Cafés and research focus groups, the structure of World Cafés facilitates participants contributing to smaller group conversations that build on each other to feed into a broader investigation of a complex topic (Brown 2005, Löhr *et al*. 2020). Thus, it may be an innovative method for bringing together different types of expertise from multiple academic disciplines and sectors of society, or a variety of perspectives, including government, academics, and practitioners.

It is recommended to have at least 12 people at a World Café, which is larger than typical focus groups, to allow for enough people to form multiple simultaneous small group conversations around different tables (Brown 2005, Löhr *et al*. 2020). Some of the key design elements are to set the context, adopt clear purposes, and expectations concerning listening and respect at the start of the World Café, to make the space convivial to emulate a real café, to encourage creativity, such as by covering the tables in paper tablecloths that people can note and draw on, and to facilitate small group conversations on specific topics at a set of tables through which people can choose to circulate (Brown 2005). World Cafés are broken up into sessions where people can join a table, contribute to a discussion, and then choose to switch to another table or stay where they are (Brown 2005). After several such sessions, the table discussions are reported back and reflected upon as a large group, to move towards consensus and the identification of priority solutions (Brown 2005). Typically mind maps or other creative notation approaches are used to depict and further generate conversations (Brown 2005).

Recchia *et al*. (2022) reviewed 25 health promotion and prevention studies that used the World Café method and concluded that the method was valuable for creating a relaxed atmosphere that encouraged collaboration and creativity, could engage a wide range of people on a common goal, could develop new rather than predetermined outcomes, and could aid prioritization of issues. The studies they reviewed focused on single sector perspectives (e.g. health professionals) and/or lived experience perspectives. We found a few examples of World Cafés that touched on social determinants of health in the literature (Bainbridge *et al*. 2012, National Academies of Sciences, Engineering, and Medicine; Institute of Medicine; Board on Population Health and Public Health Practice 2016), though we found only one that sought an intersectoral/interdisciplinary set of participants. Yonto (2020) ran a World Café to look at social mobility in rural communities in the USA, and included academics and community practitioners across the sectors of education, health, transport, and housing, as well as community members. Yonto (2020) reported successfully integrating the different sector perspectives in their World Café through diverse conversations to yield themes including on transport and community health. The current study is designed to add to this literature exploring World Cafés as a method for gaining intersectoral insights on health and health equity.

An applied example of intersectoral World Cafés on social determinants of health equity. This article provides an account of how we employed the World Café method to gather policy recommendations for how to reverse growing health inequities in Australia. We include our reflections on the strengths and weaknesses of the method. The research was driven by data indicating that health inequities in mortality, and in burden of disease, have been worsening over recent decades in Australia (Flavel *et al*. 2022a, Timonin *et al*. 2025). Our social determinants of health research had identified a wide range of structural determinants driving this trend, including increasing inequities in income, wealth, employment, education, and housing, as well as climate change and privatization and outsourcing (Musolino *et al*. 2020, Flavel *et al*. 2022a, Baum *et al*. 2025). Thus, we sought to identify which public policy changes in each of these sectors and issues would likely increase health equity in Australia. The research question guiding this article was:

How can the World Café method aid bringing together multidisciplinary and multi-sector expertise to identify solutions for health promotion issues?

## Methods

We conducted one in person World Café in each Australian state and territory (*N* = 8) between June 2024 and September 2025. Ethics was received from the University of Adelaide Human Research Ethics Committee (H-2023-099).

### Procedures

#### Inviting participants

For each World Café, we sought to invite a wide cross-section of experts from sectors relevant to the social determinants of health inequities, particularly: housing, climate change, income, wealth, labour market, public service, health care, public policy, Aboriginal and Torres Strait Islander health, disability, LGBTIQA+ people, Culturally and Linguistically Diverse people, rural and remote health, urban planning, education (primary, secondary, and tertiary), early childhood, and social services. We identified experts to invite through our existing professional networks, web searches for health and other sector non-government organizations, peak bodies including the Councils of Social Service in each state/territory, university staff directory searches for academic experts, and through snowballing, where many participants recommended other local experts to invite. Between 57 and 97 people were invited in each state/territory.

#### Preparing the space and setting the scene

Prior to the World Café, participants were sent a copy of the project’s interim report (Baum *et al*. 2025) which included an overview of the project aims, health equity data, and analysis of the social, economic, and structural determinants of health equity.

**Figure 1:**
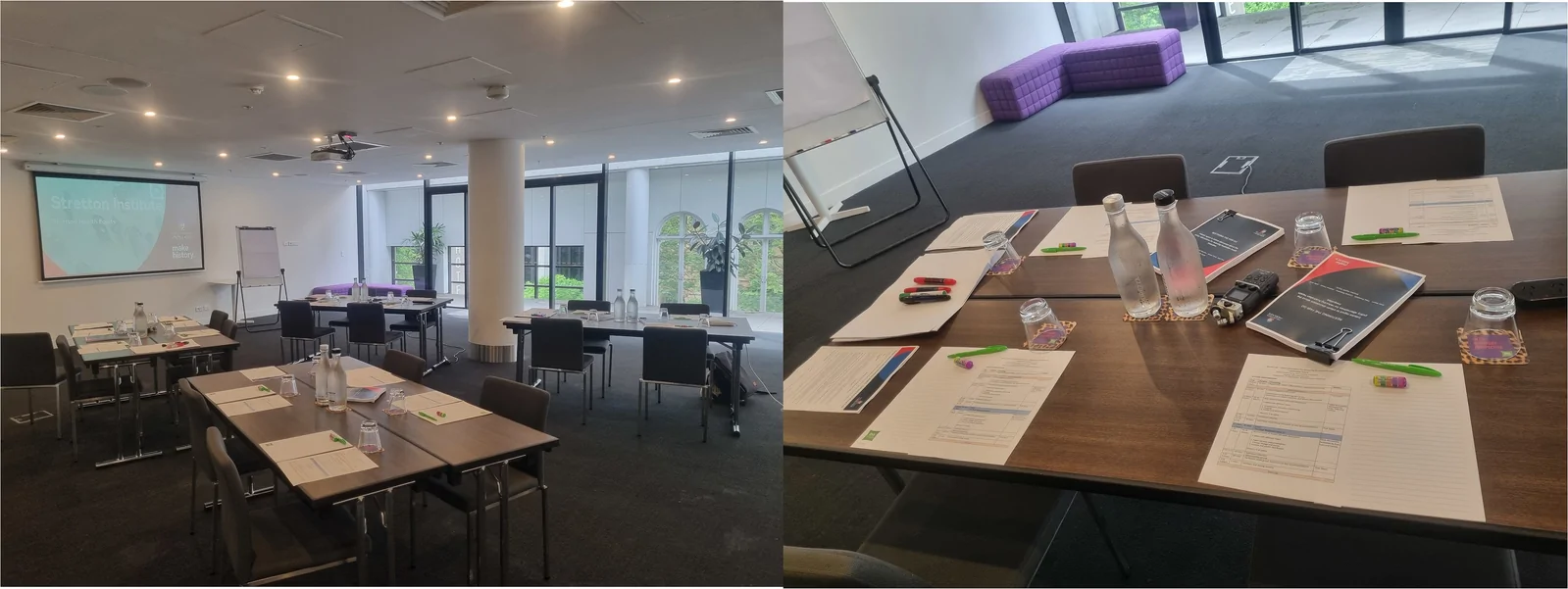
Room set up for the World Cafés.

On the day, facilitators arrived early to set up the tables with information sheets, consent forms, audio recorders, copies of the background research report, paper, and coloured pens for writing and drawing. The room set up is shown in Fig. 1.

Each World Café commenced with an informal morning tea to allow participants to meet or catch up, which helped to create a more relaxed and convivial atmosphere. This was followed by an overview presentation by author and lead researcher FB, covering: the increase in health inequities in Australia; data on key determinants of those inequities (e.g. housing, income inequities, and wealth inequities) for that state or territory; and the aim and rationale for the World Café method to develop a shared understanding of the drivers of health inequities, and to generate policy recommendations to reduce health inequities.

#### World Café facilitation

**Figure 2:**
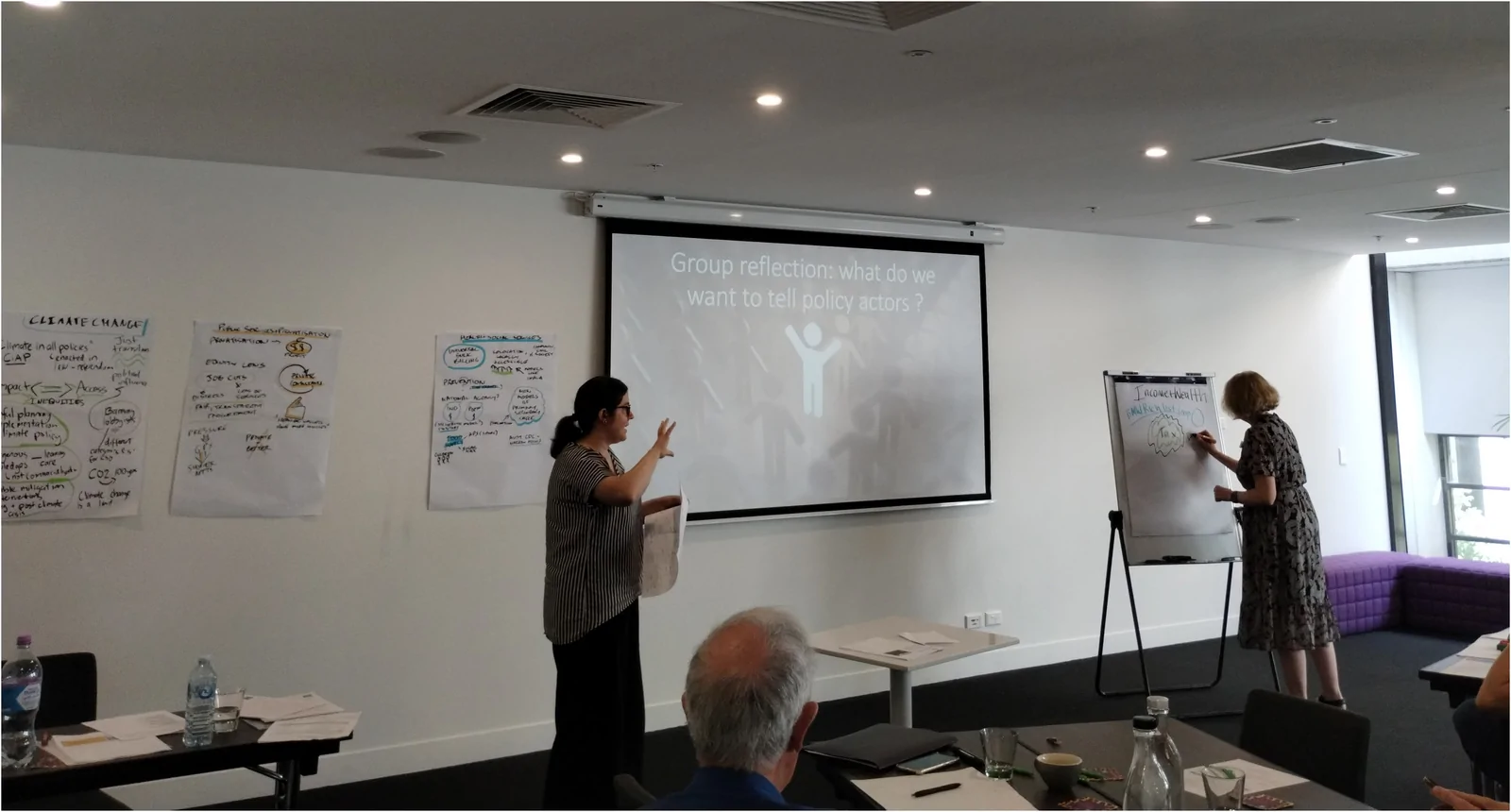
Two of the authors facilitating a feedback session in a World Café.

In the next 1.5 h session, three tables were facilitated by FB, CM, and TF, on: employment, education, and training; housing; and health and social services. The guidance given to participants was ‘to reflect on the research findings and suggest recommendations to reduce health inequities in Australia.’ The findings for each table’s topic were included in the background report and scene-setting presentation, and participants were asked if the findings resonated in their jurisdiction, and what recommendations they would propose to redress inequities. Time was called at each half hour, when participants could either move to a different table, or remain in the same conversation. At the beginning of the second and third half hour, the facilitator gave a brief summary of the conversation so far. After the session, participants reported back the key policy recommendations identified in the discussions they were a part of, and one member of the research team used visual notetaking to minute the report back. Figure 2 is a photo of one of these visual notetaking sessions. After a break for lunch provided by the research team, this process was then repeated for a second session, with tables on: public services and privatization; climate change and equity; and income and wealth distribution. This process was followed in all World Cafés except for the Northern Territory, where only two facilitators were available, so the six tables were split into three sessions of two tables each.

#### Data collection

Discussions at each table were audio recorded and transcribed, either using Microsoft Word transcription, or by a third party transcription company, and in either case then checked and corrected by a member of the research team. Given the group nature of discussions, quotes were not able to be attributed to individual experts. Participants were also provided with pens and paper to jot down notes and draw. The table facilitator also took written notes for each table discussion. The visual notes from the reports back were scanned and retained as data.

Finally, participants were requested to complete individual reflection sheets after each session, to record: what they appreciated about the conversation; what surprised them; what was missing; and what we needed more clarity on. These sheets were collected at the end of the World Café and also entered as data for analysis.

### Analysis

Transcripts were imported into NVivo, which the team used to undertake reflexive thematic analysis (Braun *et al*. 2023). A codebook was developed drawing on literature on social determinants of health inequities, the topics guiding the World Cafés, and the research questions. Themes identified during data analysis were brought to team meetings, discussed, and added to the code book. The codebook is presented in Supplementary Material S1. Coding was developed and trialled by authors CM and TF, and implemented by a research assistant, with two transcripts double coded by CM or TF as a check on coding quality, and regular meetings held to discuss and debate identified themes, findings, and approaches to coding. We found reflexive thematic analysis was a valuable tool to analyse the World Café transcripts because the way participants described problems and proposed solutions were deeply rooted in value judgements that sometimes matched those of the researchers, and sometimes conflicted. Reflexivity was key to surfacing these values. The regular team meetings and the analytical process of not just selecting solutions generated in the World Café but also understanding from the problems and solutions discussed the consistent barriers facing action on health equity, were crucial mechanisms for reflexivity.

## Results

The number of participants that attended each World Café is shown in Table 1.

**Table 1:** Participants in each state/territory World Café.

State	*N*
Tasmania	18
Victoria	24
Queensland	17
NSW	19
NT	16
SA	29
WA	24
ACT	15
Total	162

The breakdown of participants by sector and area of expertise is shown in Table 2.

**Table 2:** Characteristics of World Café participants.

	*N*
**Sector**	
Government	26
Non-government	70
Academic	60
Other	3
Not recorded	3
**Area of expertise**	
Health services/social and commercial determinants of health	89
Social services/Anti-poverty	13
Housing/Urban planning	13
Aboriginal health/affairs	10
Climate/Environment	7
Economics/Employment	5
Youth	5
Multicultural	4
Education	3
Disability	3
Public policy	3
Law	3
Older people	1
Not recorded	3

We found representatives from different sectors readily found common ground and were able to collaborate on topics to identify potential public policy and other solutions to how their sectors impacts on health inequities (‘There was a lot of agreement despite diverse starting point’, WA World Café personal reflection).

Examples of the common ground that united attendees across sectors included: how inequities were mirrored across sectors (‘it sort of surprised me how often consistent themes come up → gender inequity SES inequities across all three topics → housing but also employment + health + social services’, Victoria World Café personal reflection), the power of vested private interests, impacts of privatization (including in education, transport, housing, job service networks, and utilities), concerns with short-term and timid political responses to central societal concerns (‘Low risk appetite of policy makers’, ACT World Café personal reflection), and the growing impacts of climate change on all sectors.

We found this identification of commonalities across sectors also led to discussion of deeper, more structural drivers and proposed solutions than what may have been yielded by more sector-specific or individual research methods. For example, a recurring cross-sectoral theme around solutions was the need to have a societal debate on the values that drive our public policy decision making, e.g.:

‘We talked about culture shifting the mindsets. How do you get people to value and appreciate and understand where we will be in the future, and that this is what we’ve got to do and this is what we should be aiming for. And then how do we advocate that to our politicians and leaders who make the decisions.’ (Tasmania World Café)

Intersectoral collaboration was also frequently exhorted as a key strategy to act on social determinants of health equity, and the siloing of sectors, within and outside of government, was often named as a barrier to action on equity:

‘For me the biggest issue is siloed thinking. We all continue on our little tram tracks and on our little pathways and we say, ‘What can we do with the bit of money we’ve got?’ or, ‘What can we do within the policy space that we are permitted to work within?’ It seems to me that the equity issue, regardless of whether you want to look at it from an education perspective, a health perspective, might be a legal perspective or whatever other, they are all interrelated.’ (Tasmania World Café)

Other upstream determinants that were captured in World Café discussions included the power of commercial interests across sectors, including housing developers, the pharmaceutical industry, and fossil fuel corporations, how the public service had been hollowed out in favour of a reliance on private consultants, and how parallel private and public systems across multiple sectors including in education and health had created entrenched inequities.

**Figure 3:**
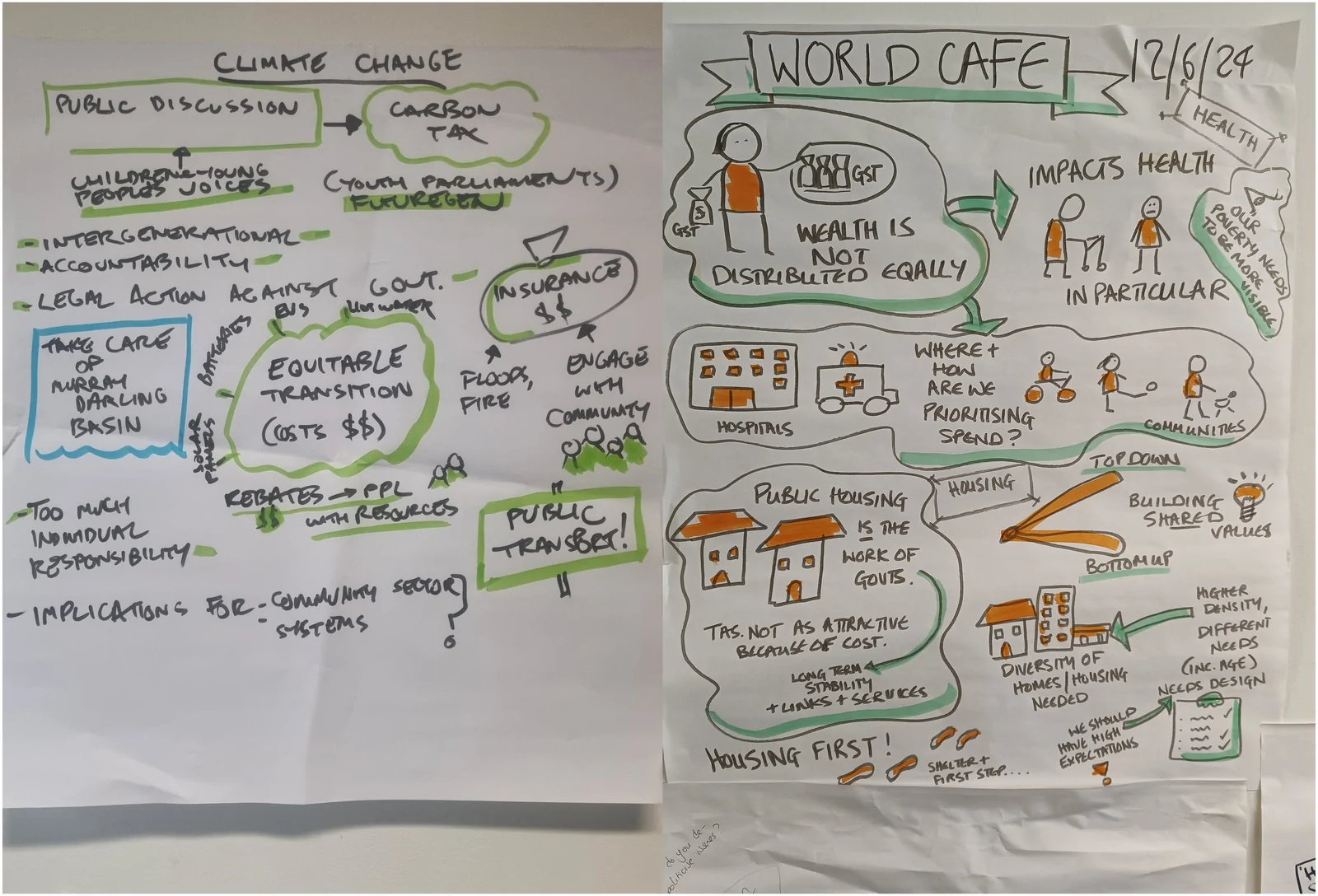
Examples of visual note taking summaries, for Climate Change (ACT World Café) and Health and Social Services (Tasmanian World Café).

The report back from each table captured using visual note taking was effective in highlighting how intertwined all the sectors and social determinants of health were, highlighting the complex systems all actors were operating within. Examples of the summary visual note taking from different sessions are shown in Fig. 3 below.

We conducted World Cafés in each state and territory to capture local jurisdictional detail on equity issues and action needed on determinants of health equity. We found there were important differences in the challenges faced by different jurisdictions. For example, the impacts of climate change varied strongly between jurisdictions (e.g. cyclones in Northern Australia, while floods were more of a concern in New South Wales and Victoria). For the Northern Territory, with the highest Aboriginal and Torres Strait Islander population proportion in Australia, issues of self-determination, overincarceration, and underinvestment in Aboriginal services were particularly salient, which, for example, drove higher rates of housing insecurity and homelessness in the Territory. Different jurisdictions also had different strengths in policy initiatives targeting equity, including subsidized public transport in Queensland, ACT leading in the transition to renewable energy, and community health centres in Victoria. However, it was also apparent that the central barriers faced, included vested commercial interests, short-term politics, and government disinvestment in crucial determinants of equity, such as public housing, were very similar across jurisdictions.

Feedback about the World Café from attendees was very positive, particularly highlighting the value of bringing different sectors together to address shared challenges:

‘How so many people have varied backgrounds shared their knowledge and expertise—so refreshing compared to working in isolation as an academic. I really valued this aspect of the workshop.’ (SA World Café personal reflection)

Participants valued ‘the diversity of ideas and experience in the group’ (QLD World Café personal reflection) and being able to contribute, e.g. ‘As an Aboriginal woman having my voice heard & valued was really appreciated.’ (SA World Café).

One useful critique was that despite the involvement of attendees from multiple different sectors, the World Café had not succeeded in capturing the complex inter-relatedness of the system of social determinants:

‘When I read the report and what I've heard today, the way that today's structure, what seems to me to be completely missing from your whole analysis, or it may be that you’ve parked it for the time being, is a complex systems view of all this. Complex systems analysis. We are bouncing around from all sorts of areas … I know we have to divide it up to think about it and all the rest of it, but at some stage one needs to bring it all back together.’ (NSW World Café).

While the report back session did offer some opportunity to bring systems and cross-sectoral perspectives together, e.g. around equitable and just transitions, which crossed sectors including employment, climate change, taxation, and housing, there was no session in the World Café to bring all the material together for a whole of system analysis. We have found that the World Cafés provided extensive material for the research team to undertake this whole of system analysis.

We also observed across the World Cafés many instances of soft benefits—of networking across sectors, people swapping contact details, and arranging future collaborations. People also reported valuing the time spent in a space that centred equity and social justice, finding it invigorating to realize the extent of shared purpose and value across sectors in a context that could be frequently demoralizing and isolating (‘The opportunity to link with colleagues across sectors & hear what other are doing, & their thoughts & reflections are always wonderful. It creates a sense of engagement & connection that we currently feel lost from’, Queensland World Café personal reflection).

## Discussion

We found World Cafés to be a valuable research method for bringing together expertise from a wide range of sectors to identify solutions to addressing the social determinants of health. Drivers of key health issues often have origins in many different sectors of society (Commission on the Social Determinants of Health 2008, Marmot and Allen 2014), and the World Cafés allow researchers to bring together this shared expertise to collaboratively analyse problems and develop solutions. Our previous research has also shown that building political will to address the social determinants of health inequities also requires reaching policy actors in government, academia, and civil society (Baum *et al*. 2022), and we found bringing these groups together also helped generate ideas and momentum to build this political will.

There has been an ongoing struggle to reorient health promotion action away from individual health behaviours and skills to address policy drivers of population health (McMahon 2022, World Health Organization 2025). Such upstream policy action holds much potential to shift persistent health inequities in the population, as they can influence the distribution of important resources for health such as income, employment, housing, safety, and access to healthy food (Baum and Fisher 2011, 2014). Downstream interventions focused on behaviour by contrast are unable to address these structural constraints and are unlikely to be effective for people with less socio-economic resources (Baum and Fisher 2011, 2014). Nevertheless, behavioural strategies continue to have enduring appeal to governments, funders, and researchers. One strong benefit we found of bringing together multiple disciplines and sectors in World Cafés to discuss inequities was that the identification of common drivers across sectors naturally pushed discussion towards upstream structural policy issues and solutions. This has informed our analysis and recommendations arising from the research. Therefore, intersectoral methods such as World Cafés may contribute to refocusing health promotion efforts towards upstream, structural drivers of health inequities.

The World Cafés required considerable effort to recruit in sectors and states and territories where we did not have pre-established networks. The analysis of the extensive transcripts and notes generated by our eight World Cafés was also resource intensive. However, we found the rich data generated by the method to be very valuable to answering our research questions on why health inequities have been increasing in Australia, and what policy changes may reverse these growing inequities. That participants also valued contributing to the World Cafés also made the research more of an exchange compared to other traditional data collection methods such as interviews that can be one-directional and extractive. Academics, civil society, and government all struggle to overcome silos, yet such collaborative opportunities are crucial if we are to address complex questions such as how to reduce health inequities (Townsend *et al*. 2020, World Health Organization 2023). Our experience suggests there is great appetite amongst all three of these sectors to think and work across siloes.

Through conducting 8 World Cafés nationwide, we learned some practical lessons for running successful World Cafés:

1. Extensive lead time is required for recruitment given the expected time commitment and administrative load of a World Café (essentially a full day out from normal employment), and given the need to iteratively identify gaps in sectors represented and expand recruitment efforts to fill these gaps.
2. Time needs to be invested for all facilitators to meet, understand deeply and have shared agreement on the World Café method, aims, and procedures, including delegating tasks such as handling the audio recorders, and information and consent sheet packs. Ongoing reflection on the best ways to support an inviting, conversational environment while managing group dynamics such as speakers dominating discussion were valuable.
3. Venues need to be carefully selected to (a) maximize geographic accessibility (e.g. located centrally or on good transport routes), (b) offer spacious, quiet environments for recording conversations, and (c) be accessible by the public. Spaces that required keycard access created difficulties for participants arriving late, or needing to come and go during the World Café, increasing the difficulty of facilitation. We found conference rooms in hotels were typically the best option to fulfil these criteria. Providing a generous morning tea, lunch, and afternoon tea was important to attract participants, to create a congenial environment, and for a sense of reciprocity. Using a hotel also meant facilitators were not over burdened with tasks such as coordination of catering.

The limitations of our work included that we struggled to attract participation from the business sector. After two attempts to invite key business peak bodies to the generic World Cafés we promoted a business specific World Café in South Australia, but failed to garner enough participants to run a World Café, instead inviting those respondents to do individual interviews. This may reflect the topic of the World Cafés (addressing health inequities) not a topic familiar to business representatives, or a priority for them to engage in, especially when no payment is offered. The goals of the World Cafés meant that we focused more on professional representatives and expertise, rather than lived experience perspectives, although some participants did bring such perspectives because many participants were in advocacy roles where they spoke from a lived experience perspective as well as a professional organizational perspective, such as disability organizations and Aboriginal community controlled organizations.

World Cafés as originally designed are inherently in-person events, and we agree with Recchia *et al*. (2022) that this format is very valuable for hosting creative and interactive discussions. However, we acknowledge the in-person nature risks excluding some people. We had at least one rural participant request funding for travel that we could not provide, and while several people with a disability attended, more may have been excluded by the in-person format. World Cafés have been conducted virtually, particularly in response to COVID-19 pandemic restrictions (McKimm *et al*. 2020, Kinney and Kinney 2024), and this may be worth exploring as an option to maximize participation, especially as McKimm *et al*. (2020) note, to foster international collaboration where an in-person event would be prohibitively costly. Kinney and Kinney (2024) did report difficulty in their virtual World Café in creating a welcoming space and sustaining conversations, suggesting some of the benefits of World Cafés may be reduced when run online. The informal interactions that occurred before, during and after the World Café which were appreciated by the participants would also not occur in an online format.

## Conclusion

World Cafés proved to be a very fruitful and informative research method for bringing experts across multiple sectors together to identify solutions to address social determinants of health equity. While resource intensive, the method yielded rich, detailed data, as well as benefits for participants, and we would suggest it is a valuable addition to a health promotion researcher’s toolkit for methods seeking structural solutions to health inequities and other health promotion topics. Such upstream focus is likely to identify opportunities to make meaningful changes to government policy and social structures to create healthier, more equitable societies, which are urgently needed as health inequities continue to worsen globally (Rad 2025).

## Supplementary Material

daag135_Supplementary_Data

## Data Availability

The data are not publicly available because this consent was not sought from participants.
