# Supplementary material for: World Cafés as a method of identifying cross-sectoral policy solutions to social determinants of health equity in Australia
Source: Health Promot Int. 2026 Sep 4;41(5):daag135. doi: 10.1093/heapro/daag135 (PMC13543679; doi:10.1093/heapro/daag135)
Supplement: Supplementary file 1 [file daag135_supplementary_data.docx]

Supplementary Material 1 – World Café Analysis Codebook

Codes

| Name | Description |
| --- | --- |
| Advocacy |  |
| Advocacy strategies |  |
| Barriers and supports for advocacy | Include barriers and difficulties associated with policy response and reform (i.e., political will) |
| Examples of successful advocacy |  |
| Narratives and Awareness | Including any references to prevailing discourses, language, ideas, concepts, and need for understanding, values, questioning assumptions, and paradigm shifts. Also contribution of media (especially social media). |
| Approaches to equity |  |
| Aboriginal and Torres Strait Islander peoples | References to Aboriginal and Torres Strait Islander peoples in terms of impact on their communities and also what we can learn from them. |
| Age | Include references to cohorts based on age (e.g., youth, middle age, older people) |
| Culturally and Linguistically Diverse, Migrants, Refugees |  |
| Disability |  |
| Disadvantaged groups - poverty | When policies and programs target disadvantage groups and/or focus on poverty, rather than taking a 'gradient' approach. Code here when this reflects the language used by the participant, or for policies designed to target a disadvantaged group, e.g. policies for people in social housing. |
| Gender | Also including considerations around parenting, family, domestic violence, and LGBTIQA+ issues under this code. |
| Gradient | The social gradient in health is a term used to describe the phenomenon whereby people who are incrementally less advantaged in terms of socioeconomic position have incrementally worse health (and shorter lives) than those who are more advantaged. As opposed to a view of the disadvantaged as a group e.g. 'people in poverty'. Code here e.g. for any discussion of existing gradient, or policies that people believe will flatten the gradient. |
| Intergenerational | Intergenerational perpetuation or exacerbation of inequities |
| Rural and Regional |  |
| Universalism | Universal policies aim to ensure equal access for all citizens to a particular good or service e.g. Medicare, Universal basic income. Code here for discussion of universalism or policies that aim to be universal, even if pointing out holes in universalism e.g in Medicare. |
| Cross cutting themes |  |
| Climate Change | Any discussion of impacts of climate change, natural disasters, policies to address climate change, or on considering equity and policies around decarbonisation, e.g. electric vehicles, solar. |
| Community building and Place | Considerations of collectivism and social cohesion; the role of community in health (including community health centres), social services, and advocacy; urban planning, design, and place-based perspectives. Will also code family into this. |
| Corporate Determinants | Explicit mention of the role of corporations and businesses (note that this code overlaps quite a bit with "power-interest", "privatisation", and "privilege - wealth"). |
| COVID-19 | Coding any references to COVID-19 pandemic, particularly in relation to lessons learned and positive examples from the pandemic response. |
| Democracy & Government | Considerations of democratic processes, and broader issues at local, state and federal government levels, and considerations of role of government. |
| Economic rationalism - Efficiency | Code here for any discussion of this as a driver of inequitable policy and ideas for addressing/countering it. |
| Individualism |  |
| Intersectoral collaboration - Silos | Including across government department and three levels of government |
| Managerialism - New Public Management | NPM an approach to public sector reform that applies private sector management techniques and business principles to public services and government organizations to improve efficiency, effectiveness, and responsiveness. Code here for any discussion of this as a driver of inequitable policy and ideas for addressing/countering it. |
| Power - interests | Interests - vested interests like industry lobbyers in Canberra, or medical power, or the power of developers in housing and urban planning. |
| Privatisation | Any discussion of privatisation of any service or function inc. transport, schools, health services, policy making, prisons etc. And any discussion of renationalisation/bringing services or functions back into public ownership. Discussion of NGOs and NFPs also included here (though not quite "privatisation" proper). |
| Privilege - Wealth | Any focus on the other end of the gradient - people having too much privilege or too much wealth - e.g. private schools generating privilege. Also social privilege in terms of social connections and leveraging those connections to get ahead (e.g., employment and nepotism). |
| Reflection Type |  |
| Change over time |  |
| Driver of inequities |  |
| Measures and Metrics | Limitations with how we measure/assess outputs or performance of various organisations and public services (e.g., in terms of KPIs). Including considerations of costs and costs that are offset by addressing health inequities. Including broader measures such as GDP. |
| Policy recommendation | Also include general policy ideas; this code may also contain some quotes pertaining to role of government and overlaps with the "Advocacy" codes. |
| Positive Practice | Discussion of a positive example of a practice reducing inequities |
| Sector |  |
| Business & Industry |  |
| Education | Includes education at all levels - early childhood, primary, secondary and tertiary. Also includes childcare. |
| Employment & Unemployment |  |
| Energy | Also including some points about water supply and water security |
| Environment |  |
| Food and Water Security | Including water security |
| Health |  |
| Housing |  |
| Social Security | All welfare policies and government income support and welfare state discussion - e.g. jobseeker, Centrelink, Robodebt, carers allowance, disability support |
| Social Services | Including disability care (NDIS) |
| Taxation & fiscal | Taxation policies, and general fiscal and economic environment and policies. Also include considerations of government investment, spending, funding. |
| Transport & Accessibility |  |
